# A Personal Tribute to Robert B. Sim with Reflections on Our Work Together on Factor H

**DOI:** 10.3390/v13071256

**Published:** 2021-06-28

**Authors:** Anthony J. Day

**Affiliations:** Wellcome Trust Centre for Cell-Matrix Research and Lydia Becker Institute of Immunology and Inflammation, School of Biological Sciences, Faculty of Biology Medicine and Health, University of Manchester, Manchester Academic Health Sciences Centre, Manchester M13 9PT, UK; anthony.day@manchester.ac.uk; Tel.: +44-161-2751495

Robert (Bob) Sim had a profound effect on almost every aspect of my approach to scientific research, acting as a mentor and moral compass through the many different stages of my career. I am indebted to Bob for his fatherly supervision, his friendship as a colleague and collaborator and his sage advice whenever it was asked for. I had the great privilege to work with Bob—one of my scientific heroes—over a period of 26 years and we published 21 primary papers and 6 review articles together. I was Bob’s second D.Phil. student (sandwiched between Vivek and Rajneesh Malhotra) and learned so much from him, not only how to design an experiment and be observant and critical of one’s results, but also about how to behave correctly as a scientist. In later years whenever I needed advice on how to handle a particular situation, Bob was always the person I turned to for his calm clear vision, integrity and sense of fair play.

I first met Bob in 1984 when I arranged to do my 10-month final year (Part II) project with him in the MRC Immunochemistry Unit, as a chemistry undergraduate at the University of Oxford. My project was focused on human complement factor H (FH) and continued experimental work Bob had done himself to generate tryptic peptides of FH for protein sequencing. Bob had recently published with Richard DiScipio a method to purify FH and had carried out initial characterisation [1]. This included determining the protein’s size at 155 kDa, its N-terminal sequence, amino acid composition and proportion of carbohydrate, as well as FH’s sedimentation coefficient and frictional ratio. These pivotal studies showed FH was a highly elongated protein and, also, that it could be proteolytically cleaved into disulphide-linked fragments without loss of its cofactor activity. 

Bob likely assigned me to continue the analysis of the primary structure of FH because of my chemistry background and this set me on a journey to become a protein chemist, which is how I still describe myself today. During the early months of my Part II, Bob patiently taught me how to handle proteins and purify peptides by ion exchange and reverse phase chromatography. He also encouraged me to read papers on the complement system and biochemistry in general, which was challenging given that I had done no biology since O’ level and knew nothing about the innate immune system. Looking back, I realise I was probably a rather needy student and Bob was exceptionally tolerant (then and later on) of my endless questions. Over the years, I must have spent tens of hours (maybe hundreds!) standing at Bob’s office door, with him sitting surrounded by files and piles of paper answering my questions, giving me advice/encouragement or just chatting (see Figure 1).

Working closely with Tony Willis, amino acid sequence information was generated on the tryptic peptides of FH I had purified under Bob’s guidance [2]. One such peptide was chosen as the basis to design a mixed 17-base long oligonucleotide probe (consisting of 32 sequences) for screening of a liver cDNA library. Bob and I synthesised this degenerate probe together using a Cruachem Manual Module, where one had to inject the various synthesis regents every few minutes and with strict timing of incubations. The whole process took about 12 hours (if I remember correctly) and when I made an error (about 9 hours in!), Bob just said not to worry and that we would try again tomorrow. This illustrates Bob’s kind, supportive and forgiving nature. I also learnt a valuable lesson that mistakes are acceptable if you admit/recognize they have occurred and that you do not make a habit of them! I am pleased to say that the synthesis went smoothly the second time around and led ultimately to the isolation of the first cDNA clones for human FH by Jean Ripoche, an EMBO postdoctoral fellow in Bob’s lab [2]. 

In the summer of 1985 Bob persuaded Rodney Porter (the Director of the Immunochemistry Unit) to invite me to the party at his house to mark his retirement as the Whitely Professor of Biochemistry; it should be noted that it was very unusual for undergraduate students to be invited to such events and I think Bob went out on a limb to include me. I remember this as an amazing experience. For example, I found myself in the same room as Caesar Milstein, Rodney Porter and Fred Sanger (who between them had been awarded 4 Nobel prizes), although I was far too timid to say anything! The retirement party was at the end of a Biochemical Society conference in Oxford to celebrate Professor Porter’s career which was also my first opportunity to present a poster on some results from my Part II project; an accompanying paper (also my first) was published in *Biochem. Soc. Trans.* [3].

I continued working under Bob’s excellent supervision as a D.Phil. (PhD) student. This was funded by a SERC CASE studentship, with Celltech Ltd. as the industrial partner and Tim Harris as the CASE supervisor. I feel very fortunate that Bob was successful in obtaining funding and that I was able to continue to develop my research and transferable skills under his influence. As a fallback position Bob had encouraged me to contact/visit other potential supervisors who he suggested I would do well with, and I was offered a PhD place in Aberdeen; but I happily chose instead to stay on with Bob in Oxford.

My D.Phil. project was focused on the continuation of the characterisation of human FH. As part of this I spent many months at Celltech in Slough learning the relatively new dideoxynucleotide chain termination sequencing method (devised by Fred Sanger) and used this to sequence one of the cDNA clones isolated previously (termed R2a). This clone was found to encode the C-terminal 657 amino acids of FH [4]. Working with Jean Ripoche (back in Bob’s lab in Oxford), we completed the cDNA sequencing of FH, based finally on three overlapping clones [5]. This work revealed that human FH was 1213 amino acids in length with an 18-residue leader sequence, where the mature protein was arranged into 20 homologous units now called Complement Control Protein (CCP) modules (also referred to as Short Consensus Repeats or Sushi domains) as had been found previously for mouse FH. The cDNA sequencing work [5] also identified that the human complement factor H (*CFH*) gene was likely undergoing alternative splicing leading to truncated forms, which were further characterised by Jean Ripoche in a collaborative study between Bob and Marc Fontaine at INSERM [6]. Sequencing of human FH at the protein and cDNA levels identified a tyrosine/histidine polymorphism (now referred to as Y402H) within the 7th CCP module [5,7], which was concluded to represent a sequence difference between the two most abundant charge variants of FH (FH1 and FH2). 

Although only 4 pages in length, the Day et al., 1988 publication [7] was a landmark paper for me, being written near the end of my D.Phil., with Bob suggesting that I write the paper and take responsibility for correspondence with the journal. This again illustrates Bob’s generosity and how he was always aiming to bring out the best in his students and help them develop independence. Another example of this was how Bob involved me in other studies, including the assignment of *CFH* to human chromosome 1q [8], which used as a probe the R2a clone that I had characterised earlier [4], and the sequencing of human and *Xenopus* Factor I [9,10], with my contribution to the latter papers being the computer-based analysis of the proteins’ modular structures. This is something I had become very interested in during the analysis of the FH sequence and encouraged to pursue by Bob. This interest also led to the initiation of collaboration between Bob and Iain Campbell’s group in Oxford that resulted in the first 3D structure for a CCP module, i.e., the 16th CCP of FH [11,12]. This collaboration between Bob and Iain continued and resulted in the subsequent NMR structures of the 5th CCP of FH [13] and a pair of FH CCP modules [14]. Moreover, this formed part of Bob’s long-standing interest in protein structure, for example, his working collaboratively with Stephen Perkins to generate solution structures based on X-ray and neutron scattering for FH and other complement system proteins, e.g., C3, C4, Factor D and Factor I [15,16,17,18,19], as well as other studies [20,21,22,23]. Even after I left Bob’s group (in 1988) and the Immunochemistry Unit (in 1991), he generously included me in several collaborative projects with his lab [24,25,26] and as an author on review articles (e.g., [27,28]), which was very helpful to me in the development of my CV. Moreover, I am sure it is thanks to Bob I have become a serial collaborator having learnt the value of working as part of a larger team and how to keep relationships in good repair. Figure 2 shows Bob in conversation with Danish colleagues and collaborators at a meeting in Aarhus.

At the end of my D.Phil. I was awarded the Schorstein Research Fellowship in Medical Science (from the University of Oxford) to continue working on the tertiary structure of FH with Bob. This was a two-year appointment, however, 7-months into the project I resigned in order to work on amylin with Garth Cooper, a peptide hormone that had recently been “discovered” in the Immunochemistry Unit, and which Bob was closely involved in [29]; this was a particularly exciting prospect since the La Jolla-based company, Amylin Corporation (later Amylin Pharmaceuticals), had just been founded (by Garth) to develop a new treatment for type I diabetes. My stopping working with Bob on FH was an extremely difficult decision and I had agonised about it for more than a week before finally summoning the courage to talk to Bob. Of course, typical of Bob, he was very understanding and said he thought I would do well either staying working on FH or moving onto the amylin work and I should go with what I thought was the best opportunity. In hindsight Bob must have been upset that I gave up the FH work (and with my lack of loyalty), but he never showed it at the time or later on in our relationship. When I have had similar experiences, as a supervisor myself, I have tried to be guided by the gracious way that Bob handled the situation but am not sure I could match the same level of kindness or dignity!

After working on amylin for a few years in the Immunochemistry Unit I obtained an Arthritis Research Campaign (ARC; now Versus Arthritis) five-year Fellowship to work with Iain Campbell on the NMR structure of L-selectin (a protein containing CCP modules and a C-type lectin domain). I started the fellowship in October 1991 and in early 1992 Bob drew my attention to a paper just published on human TSG-6 [30]; I was working on the floor below, so we regularly got the chance to talk. Bob thought this inflammation-associated protein looked interesting and suggested it might be a good “side project” for my fellowship, especially since it contained a CUB module (like those found in complement C1r and C1s [31], for which no structures were yet available. This seemed like a good idea, so I started working on the TSG-6 CUB module and, also, on its Link module since no structure existed for this domain either. The rest as they say is history. Over the last 29 years, TSG-6 has evolved as my main research interest, and hyaluronan and other glycosaminoglycans (GAGs) (to which TSG-6 binds) have also become a major focus of my work (see [32]). Importantly, while my L-selectin project never took off, working closely with a visiting scientist in Iain Campbell’s group, led to an NMR structure of the TSG-6 Link module [33]; Bob was acknowledged on a proceeding paper, describing the expression of “Link_TSG6” [34] for helpful discussions and critical reading of the manuscript. We did eventually determine the structure of the TSG-6 CUB module, published in 2015 [35]; tenacity is another skill I developed working with Bob!

The paper in *Cell* [33], was no doubt responsible for the renewal of my ARC Fellowship and grant successes over following years. Furthermore, when Duncan Campbell (one of the group leaders in the Immunochemistry Unit) moved to Cambridge in 1998, my developing work on TSG-6 landed me a Senior Scientist position in the Unit as Duncan’s “replacement”. I am certain I would not have been successful without Bob’s support and his belief in the potential of my research. Importantly, my wife, Caroline Milner, who worked in Duncan’s group (as an ARC Research Fellow) remained in the Immunochemistry Unit and initially we shared a (very small) office and lab space, which was the impetus for us to start to collaborate. Caroline and I still work together and jointly run a lab in Manchester (see below).

For me it was wonderful being back in the Immunochemistry Unit and having Bob as a colleague, e.g., to bounce ideas off and continue to get his advice on various things. In 2003, Bob and I took on the joint supervision of a D.Phil. student, Simon Clark (funded by an MRC Studentship), to work on a project investigating FH’s GAG-binding properties. This very much brought together Bob’s expertise on FH with my GAG knowledge, and our aim was to understand how FH-GAG interactions contribute to host recognition and protection from complement attack. Here we focused on the CCP6-8 region of FH, which we identified computationally to contain a binding site for heparin [36], because the role of CCP19-20 in self/non-self-recognition was better characterised. This turned out to be a fortunate decision! Simon had already produced recombinant CCP6-8 protein and started on its functional characterisation when in 2005 three papers were published (in an edition of *Science*) showing that the Y402H polymorphism in the *CFH* gene (see above) was a major risk factor for Age-related Macular Degeneration (AMD). We were able to rapidly mutate the polymorphic residue (amino acid 384 in the mature protein) to generate CCP6-8 proteins with either a histidine or tyrosine at the 402 position and show that this sequence change has a large effect on the specificity of heparin binding. The resulting paper [36], on which Bob and I were co-corresponding authors was the first publication showing that the Y402H polymorphism caused a functional effect, likely affecting the binding of FH to polyanionic patterns on host cells/surfaces, and thereby potentially influencing complement activation, which had been implicated in the pathology of AMD. Co-supervising a D.Phil. student with Bob was a great experience and it was fantastic to return to researching FH and the complement system, which are topics I still work on today. 

A collaborative study between Bob and me, with Susan Lea’s group (in Oxford) and Paul Barlow and Dusan Uhrin (in Edinburgh), involving X-ray crystallography and NMR spectroscopy, provided a molecular basis for the Y402H specificity change and further characterised the heparin-binding site of CCP6-8 [37]. With Stephen Perkins (UCL) we also identified that the 402H (disease-associated) allotype may self-associate more readily than the 402Y variant and that there was a small conformational change in the CCP6-8 region (in both allotypes) on binding to heparin [38]. In addition, a collaboration with Anna Blom’s group (in Lund) revealed differential binding of the 402H and 402Y CCP6-8 constructs to C-reactive protein, DNA and necrotic cells, providing additional insight on how the Y402H polymorphism could contribute to AMD [39]. 

Because of the impending closure of the Immunochemsitry Unit by the MRC (on Ken Reid’s retirement as Director in 2008), in October 2005 I took up a Chair at the University of Manchester but continued to work in Oxford as a visitor until September 2006. At this point Caroline Milner and I moved our lab (and young family) to Manchester; many group members, including Simon Clark who had just completed his D.Phil., moved with us. Bob and I continued to collaborate (now at a distance) on the role of FH in AMD with the use of human eye tissue; this work involved a colleague in Manchester, Paul Bishop (a clinician and matrix biologist) and Simon Clark as a postdoc. This led to a paper [40], showing that the Y402H polymorphism in the CFH gene greatly affects FH binding to sites within human macula. In particular, the AMD-associated 402H allotype bound less well than the 402Y variant to heparan sulphate and dermatan sulphate GAGs within the Bruch’s membrane, which forms part of the outer blood-retinal barrier and is where AMD pathology develops. We proposed that the impaired binding of the 402H form of FH would result in overactivation of the complement system, leading to local inflammation that would contribute directly to the development and progression of this major form of vision loss. Simon Clark and I have continued working together on AMD and in 2019 Simon took up a Professorship at the Institute for Ophthalmic Research, Eberhard Karls University of Tübingen, which Bob was very pleased about. We currently have a joint PhD student working on GAG-binding proteins and the complement system, thus continuing Bob’s scientific legacy.

Clark et al. (2010) [40] was to be Bob’s and my last paper together as co-authors however, there was also a US patent granted in 2010, on which we were two of the co-inventors (US7829301B2), resulting from the work carried out during Simon Clark’s D.Phil. Bob, of course, continued to research other aspects of FH that, along with additional studies on FH not discussed above, are covered in other articles published in this special issue of *Viruses* [41,42,43,44,45,46,47].

Bob’s style has influenced so many things that I do: for instance, how I supervise my own PhD students, how I act as a collaborator and how I write. I have adopted Bob’s method of providing detailed hand-written comments on drafts of thesis chapters, literature reviews, etc., with examples of how sentences should be written. When I arrived in the Immunochmemisty Unit my writing ability was poor (largely due to being dyslexic) and Bob’s patient tutoring helped immensely with my development in this area; I learnt from Bob (by example) that providing training in scientific writing was a key duty/responsibility of a PhD supervisor. Bob also provided great feedback when practising talks and I remember that he stopped me saying “this *just* shows” since it undermines the data/conclusions being presented; this is something I correct in my team to this day!

One thing not yet mentioned was Bob’s seemingly encyclopaedic knowledge of the literature. Countless times over the years I have asked Bob if knew anything about an obscure topic and he would invariably say that he did not know very much and then proceed to cite several pertinent papers. This has never ceased to amaze me. Bob read widely and had an exceptional memory and as noted above, I am so grateful to him for bringing the paper on TSG-6 to my attention. Bob, as said already, was a kind and encouraging person, but he also could be very firm when he considered this was necessary. I still remember the hand-written letter to me (left on my desk) during my D.Phil. when I was spending too much time in the Wolfson College bar and was tardy getting into the lab in the morning. Certainly, a short sharp shock when used sparingly can be most effective! I also learnt some other tricks from Bob, in particular, that inertia is sometimes a good way to deal with *difficult* issues. One thing I never got to grips with was Bob’s suggested remedy for a bad cold: a shot of whiskey in a pint of Newcastle Brown Ale—definitely a kill or cure concoction!

As well as being a great supervisor and colleague, Bob was also wonderful company and a lot of fun. Everyone in the Immunochemistry Unit used to go to the pub together on a Friday evening after work and over a pint or two I got to know Bob socially. Moreover, Bob and Edith (Figure 3) put on great lab parties at their house as well as organising picnics and punting trips, etc. At Unit Christmas parties and other gatherings if there was any dancing going on (in particular of the Scottish Country variety!), then Bob and Edith would be on the dance floor for hours. 

Writing this piece has brought back so many happy memories of working for and with Bob at different stages of my career. Although, not directly collaborating with Bob over the last 10 years, I have enjoyed meeting up with him at conferences and on other occasions and conversing with him by email or phone every now and then. I feel exceptionally fortunate to have known him and greatly valued his friendship. Bob will be very sorely missed.

## Figures and Tables

**Figure 1:**
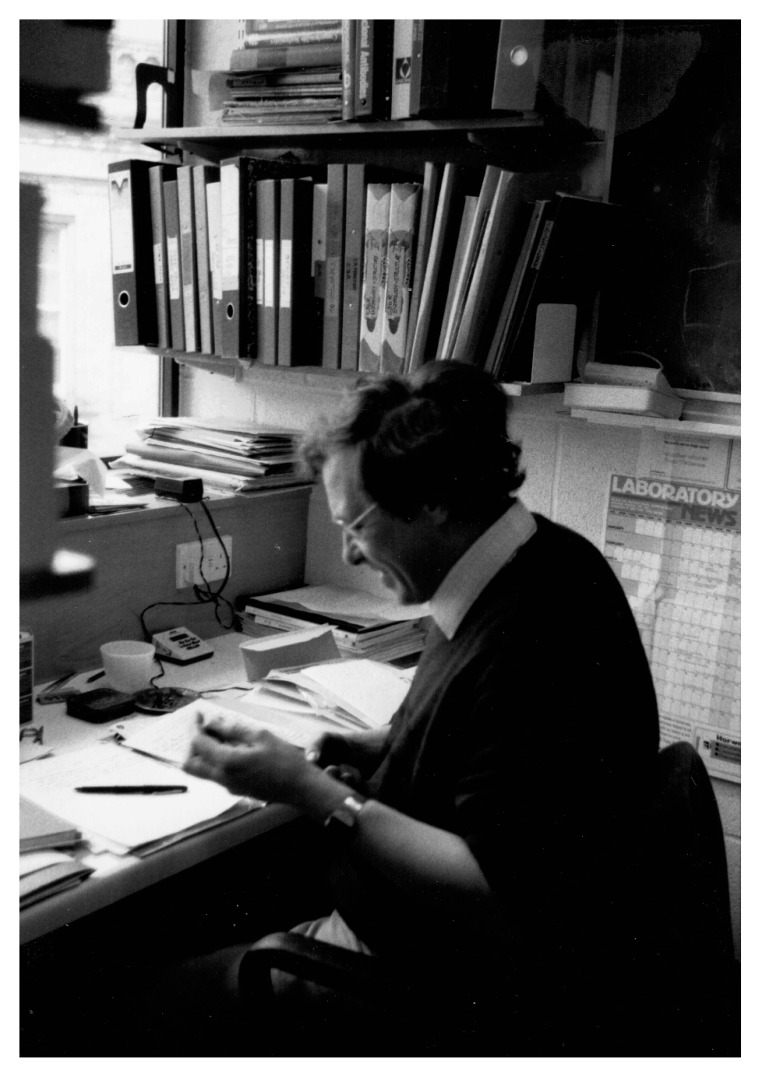
Bob Sim in his office within the MRC Immunochemistry Unit, Department of Biochemistry, University of Oxford in 1986. Copyright Tony Day.

**Figure 2:**
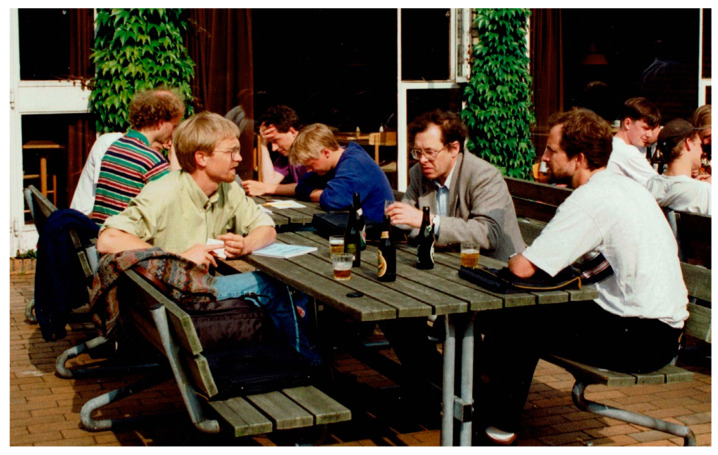
Bob Sim talking to Uffe Holmskov and Steffen Thiel at the 8th Annual Meeting of the Scandinavian Society for Immunology in Aarhus in 1993. Copyright Tony Day.

**Figure 3:**
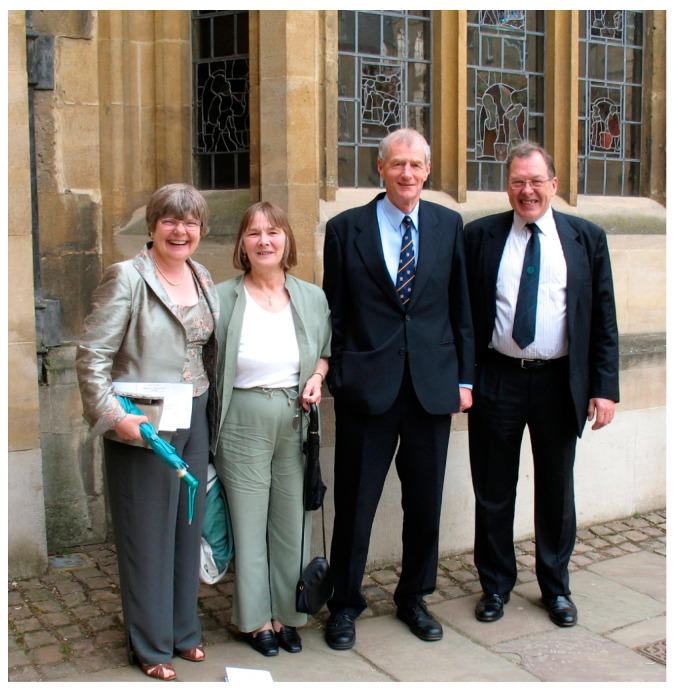
Bob and Edith Sim with Ken and Margery Reid in 2009 on the occasion of Tony Willis being awarded an Honorary MA from the University of Oxford. From left to right: Edith, Margery, Ken and Bob. Copyright Tony Day.
